# Ectopic RING activity at the ER membrane differentially impacts ERAD protein quality control pathways

**DOI:** 10.1016/j.jbc.2023.102927

**Published:** 2023-01-19

**Authors:** Adrian B. Mehrtash, Mark Hochstrasser

**Affiliations:** 1 Department of Molecular, Cellular, & Developmental Biology, Yale University, New Haven, Connecticut, USA; 2 Department of Molecular Biophysics & Biochemistry, Yale University, New Haven, Connecticut, USA

**Keywords:** protein degradation, endoplasmic-reticulum-associated degradation (ERAD), Doa10, retrotranslocation, ubiquitin, ubiquitin ligase, ubiquitin-conjugating enzyme, endoplasmic reticulum, yeast, protein quality control, CHX, cycloheximide, ERAD, endoplasmic reticulum–associated degradation, INM, inner nuclear membrane, SUS, self-ubiquitylating substrate, TM, transmembrane helix, Ub, ubiquitin

## Abstract

Endoplasmic reticulum-associated degradation (ERAD) is a protein quality control pathway that ensures misfolded proteins are removed from the ER and destroyed. In ERAD, membrane and luminal substrates are ubiquitylated by ER-resident RING-type E3 ubiquitin ligases, retrotranslocated into the cytosol, and degraded by the proteasome. Overexpression of ERAD factors is frequently used in yeast and mammalian cells to study this process. Here, we analyze the impact of ERAD E3 overexpression on substrate turnover in yeast, where there are three ERAD E3 complexes (Doa10, Hrd1, and Asi1-3). Elevated Doa10 or Hrd1 (but not Asi1) RING activity at the ER membrane resulting from protein overexpression inhibits the degradation of specific Doa10 substrates. The ERAD E2 ubiquitin-conjugating enzyme Ubc6 becomes limiting under these conditions, and *UBC6* overexpression restores Ubc6-mediated ERAD. Using a subset of the dominant-negative mutants, which contain the Doa10 RING domain but lack the E2-binding region, we show that they induce degradation of membrane tail-anchored Ubc6 independently of endogenous Doa10 and the other ERAD E3 complexes. This remains true even if the cells lack the Dfm1 rhomboid pseudoprotease, which is also a proposed retrotranslocon. Hence, rogue RING activity at the ER membrane elicits a highly specific off-pathway defect in the Doa10 pathway, and the data point to an additional ERAD E3-independent retrotranslocation mechanism.

Endoplasmic reticulum–associated degradation (ERAD) is a branch of the ubiquitin (Ub)-proteasome system where proteins are degraded at the ER, a continuous membrane system that includes the membranes of the nuclear envelope (1). In yeast, there are three transmembrane RING-type Ub ligase (E3) complexes involved in ERAD: the canonical Doa10 and Hrd1 complexes as well as the Asi complex (2, 3, 4, 5). The Hrd1 complex degrades ERAD substrates with misfolded elements in the ER lumen (ERAD-L) or membrane (ERAD-M) but is largely excluded from the inner nuclear membrane (INM) (6). The Asi complex, which is not conserved beyond yeast, localizes exclusively to the INM and degrades ERAD-M substrates in this compartment (5, 7). The Doa10 complex localizes throughout the ER membrane, including the INM, and primarily degrades substrates with misfolded cytoplasmic/nucleoplasmic elements (ERAD-C), although a few Doa10 ERAD-M substrates are known (6, 8, 9).

All the ERAD E3s in yeast require the Ub-conjugating enzyme (E2) Ubc7 and Cue1, a Ubc7 activator, for substrate ubiquitylation (2, 5, 10). Doa10-mediated ubiquitylation also requires the E2 Ubc6, which attaches an initial Ub to a substrate that is extended by Ubc7 to form a poly-Ub chain (11). Membrane and luminal ERAD substrates must undergo retrotranslocation into the cytoplasm for proteasomal degradation (1). Several proteins have been proposed to serve as retrotranslocation channels, including the ERAD E3 complexes and Derlin rhomboid pseudoproteases such as yeast Dfm1 (12, 13, 14). The ATPase Cdc48 (p97 in mammals) generates the mechanical force needed for membrane extraction (15, 16). In mammals, ERAD substrates are likely recognized and processed in a similar manner, but the number of ER-resident RING-type E3 ligases involved in ERAD has expanded significantly (1, 17).

Overexpression of ERAD components is commonly used in yeast and mammalian cells to study different aspects of ERAD. For instance, ERAD E3 overexpression has facilitated substrate identification or complementation analysis (18, 19, 20). It has also been used in yeast studies to bypass the requirement of certain ERAD components and to purify proteins for structural and biochemical analyses (13, 21, 22). Upregulation of ERAD components also occurs in response to ER stress, and increased levels of ERAD E3s have been linked to cancer (23, 24, 25, 26). Despite this, the impact of ERAD E3 overexpression on ER protein homeostasis is unclear.

Here, we employ overexpression analysis to study the Doa10 ERAD pathway. Overexpression of several Doa10 mutants specifically inhibits the turnover of soluble and membrane ERAD-C substrates. This stems specifically from elevated RING activity at the ER membrane, which causes the Ubc6 E2 to become limiting for Doa10 substrate turnover. Unexpectedly, overexpression of membrane-anchored Doa10 or Hrd1 RING domains alone causes depletion of the transmembrane Ubc6 in the absence of all ERAD E3s and Dfm1. These constructs will therefore be useful tools for studying ERAD E3-independent retrotranslocation.

## Results

### Overexpression of Doa10 mutants inhibits ERAD-C substrate turnover

**Figure 1:**
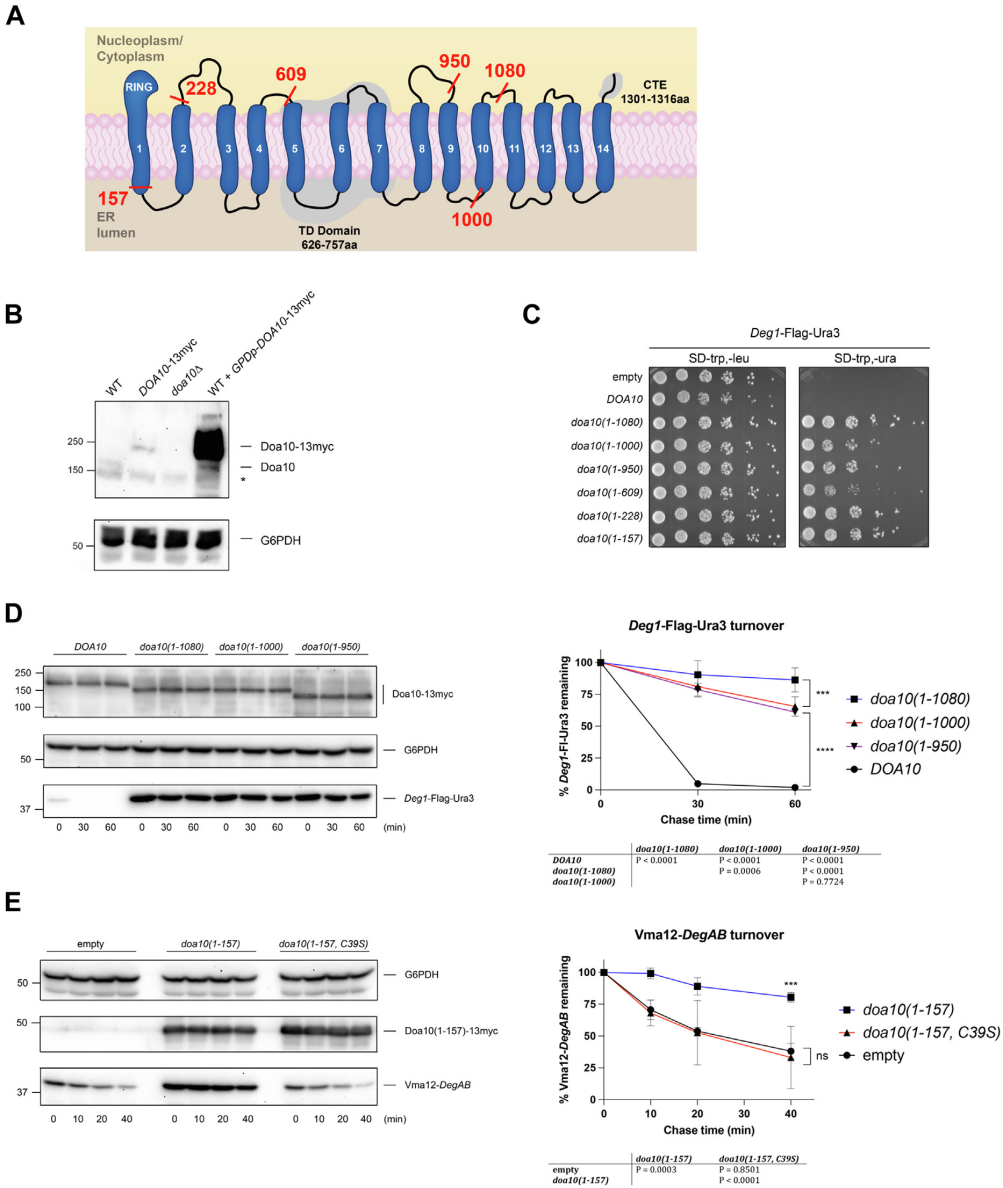
**Overexpression of Doa10 mutants inhibits ERAD-C substrate turnover.***A*, membrane topology of *Saccharomyces cerevisiae* Doa10 depicting C-terminal truncation sites and highlighting three conserved regions important for Doa10 function: the N-terminal RING, TEB4-Doa10 (TD) domain, and C-terminal element (CTE). *B*, anti-Doa10 immunoblot analysis of strains MHY500 (WT), MHY8655 (Doa10-13myc), MHY1685 (*doa10Δ*) with empty vector or MHY500 with p414GPD-Doa10-13myc. Molecular size markers (in kDa) are shown at *left*. *C*, degradation-sensitive yeast growth assays using the soluble Doa10 reporter *Deg1*-Flag-Ura3 expressed from p415MET25. Serial dilutions of MHY500 cells transformed with p414GPD expressing the indicated *doa10* alleles were spotted on SD-trp-leu (control) and SD-trp-ura (growth selection). This experiment was performed twice. *D*, cycloheximide (CHX)-chase analysis of *Deg1*-Flag-Ura3 turnover with Doa10 mutant overexpression. Experiments were performed in MHY500 transformed with p415GPD-*Deg1*-Flag-Ura3 and p414GPD expressing the indicated *doa10* alleles. Following addition of CHX, cells at the indicated times were lysed and analyzed by anti-Flag, anti-Myc, and anti-G6PDH immunoblotting. The graph (*right panel*) represents data as mean ± SD from three experiments. Band intensities were normalized to the G6PDH loading control. Molecular size markers (in kDa) are shown at *left*. *E*, CHX-chase analysis of Vma12-*DegAB* turnover. Vma12-*DegAB* turnover was analyzed as in (*D*) except MHY500 was transformed with p416GPD-Vma12-*DegAB* and the indicated p414GPD plasmids. Molecular size markers (in kDa) are shown at *left*. ERAD, endoplasmic reticulum–associated degradation. ∗*p* < 0.05; ∗∗∗*p* < 0.001; ∗∗∗∗*p* < 0.0001.

Previous studies identified several regions in Doa10 that are important for its function (2, 8, 14, 27, 28). To gain further insight into the function of these regions, we overexpressed a series of Doa10 truncation mutants (Fig. 1*A*). We reasoned the overexpression of certain mutants might have a dominant-negative effect, potentially by sequestering substrate or cofactors from WT Doa10. To this end, C-terminal truncations of Doa10 were overexpressed from the strong *GPD* (*TDH3*) promoter, enabling expression well above endogenous levels (Fig. 1*B*) and monitored Doa10-mediated turnover of *Deg1*-Flag-Ura3 using a degradation-sensitive growth assay (8). Based on this indirect assay of protein turnover, all the tested, overexpressed truncations stabilized *Deg1*-Flag-Ura3, including the small *doa10(1-157)* mutant (Fig. 1*C*). Notably, this latter mutant lacks the minimal cofactor-binding region of Doa10, spanning transmembrane helices (TMs) 1-9, including the TEB4-Doa10 (TD) domain, which likely represents the substrate-binding site of Doa10 (8, 14, 27). Only the catalytic RING-CH domain and TM1 are present in the *doa10(1-157)* fragment.

We performed cycloheximide (CHX)-chase analysis of the soluble *Deg1*-Flag-Ura3 protein to directly assess its turnover kinetics. In agreement with the growth assays, overexpression of the Doa10 mutants that enhanced growth on media lacking uracil also strongly stabilized *Deg1*-Flag-Ura3 (Fig. 1*D*). Of note, the *doa10(1-1000)* mutant, which can bind Doa10 cofactors, and the *doa10(1-950)* mutant, which cannot (14), similarly inhibited *Deg1* turnover (Fig. 1*D*).

Overexpression of these dominant-negative mutants also inhibited degradation of Vma12-*DegAB*, a membrane ERAD-C substrate of Doa10 (29) (Fig. 1*E*). While *doa10(1-157)* blocked Vma12-*DegAB* degradation, its turnover was unaffected by the overexpression of *doa10(1-157, C39S)*, which bears an inactivating RING mutation (2). Therefore, Doa10 RING catalytic activity was required for the inhibitory effect. These results also indicated that Doa10 mutant overexpression inhibits the turnover of both membrane and soluble ERAD-C substrates.

### Ectopic Doa10 or Hrd1 RING activity at the ER inhibits ERAD-C

**Figure 2:**
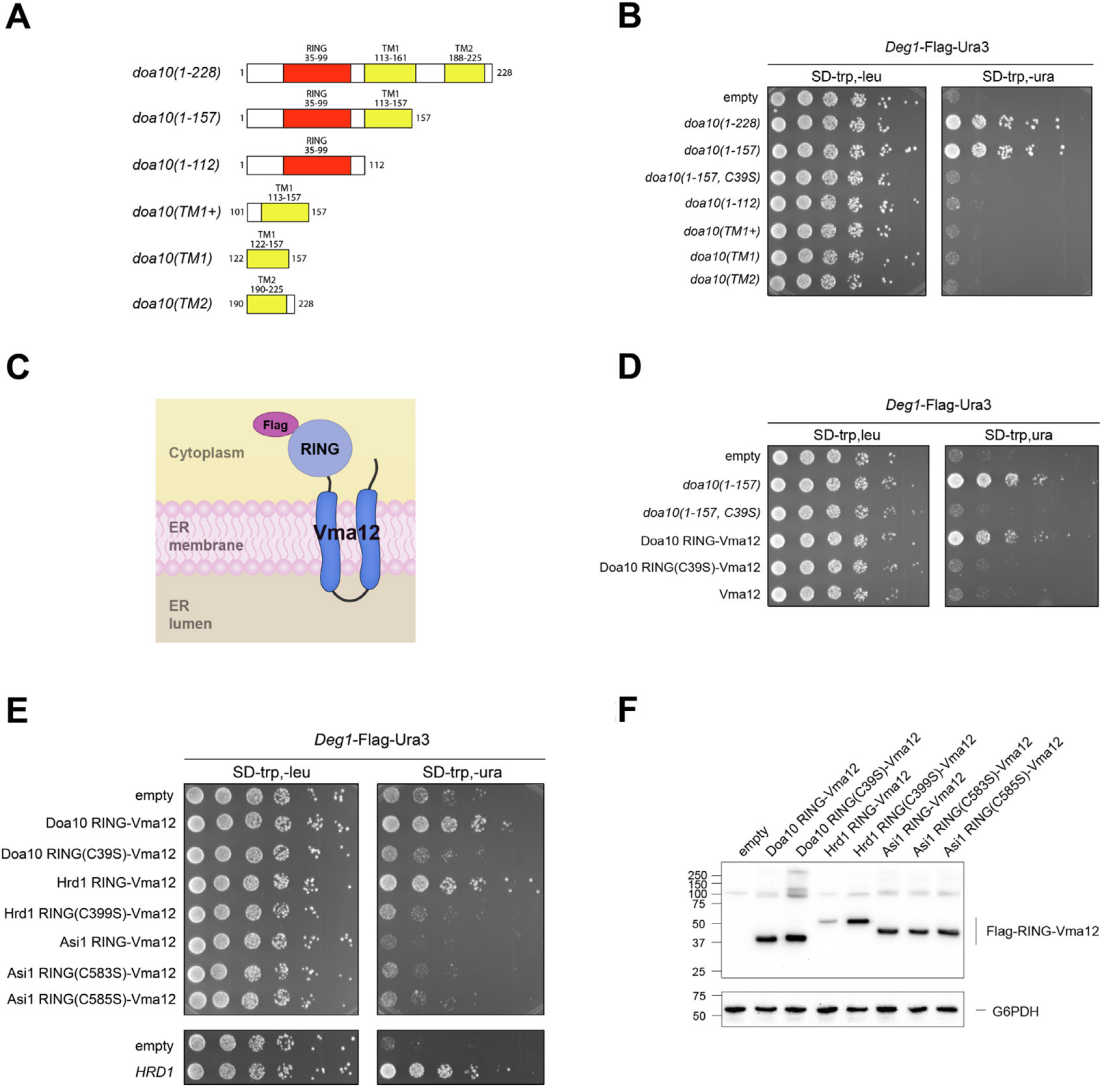
**Ectopic Doa10 or Hrd1 RING activity at the ER inhibits ERAD-C.***A*, diagram of the Doa10 mutants analyzed in panel B, highlighting the RING (*red*) and TMs (*yellow*). *B*, growth assays as in Figure 1*C* with the indicated p414GPD-based *doa10* alleles. *C*, schematic depicting the chimeric RING-Vma12 proteins used in panels *D*–*F*. These proteins contain the Doa10 RING (residues 1-112), Hrd1 RING (320-531), or Asi1 RING (473-624) fused to full-length Vma12. *D*, growth assays as in B with the indicated *doa10* alleles. *E*, growth assays with MHY500 cells expressing the indicated chimeras or full-length Hrd1. *F*, expression analysis of the chimeric RING-Vma12 proteins from (*E*). The RING-Vma12 proteins were analyzed by anti-Flag blotting. Molecular size markers (in kDa) are shown at *left*. ERAD, endoplasmic reticulum–associated degradation; TM, transmembrane helix.

To explore the mechanism by which Doa10 mutant overexpression inhibits ERAD-C turnover, we generated several additional deletion derivatives within the N-terminal 228 residues of Doa10 (Fig. 2*A*); all showed comparable expression (Fig. S1). Overexpression of Doa10 TM1 or TM2 alone or the RING-CH domain alone did not stabilize *Deg1*-Flag-Ura3. Doa10 RING activity was also required for the dominant-negative effect measured by this assay, as introducing the inactivating C39S mutation into *doa10(1-157)* prevented *Deg1*-Flag-Ura3 stabilization (Fig. 2*B*). Together, these results imply the dominant-negative effect of *doa10(1-157)* requires an active RING domain tethered to the ER membrane.

To test this idea, we made a chimera containing the Doa10 RING domain (residues 1-112) attached to Vma12, an unrelated ER membrane protein commonly used for ER membrane tethering (15, 29, 30) (Fig. 2*C*). Overexpression of this chimera, Doa10-RING-Vma12, inhibited *Deg1*-Flag-Ura3 turnover, indicating the dominant-negative effect stems from elevated Doa10 RING activity at the ER membrane and did not require Doa10 TM1 per se (Fig. 2*D*). We next tested whether ectopic RING activity at the ER membrane from other ERAD E3 ligases could also inhibit ERAD-C turnover; specifically, we made RING-Vma12 proteins that contain either the Hrd1 or Asi1 RING. Overexpression of Hrd1-RING-Vma12 or full-length Hrd1 inhibited *Deg1*-Flag-Ura3 turnover, and the inhibitory effect required an active Hrd1 RING (Fig. 2*E*). Notably, Hrd1-RING-Vma12 protein levels are much lower than the other RING-Vma12 proteins, including the ineffectual Asi1-RING-Vma12 (Fig. 2*F*). These results indicate that either increased Doa10 or Hrd1 RING activity at the ER membrane inhibits ERAD-C.

### Ectopic RING activity at the ER does not broadly inhibit ERAD

**Figure 3:**
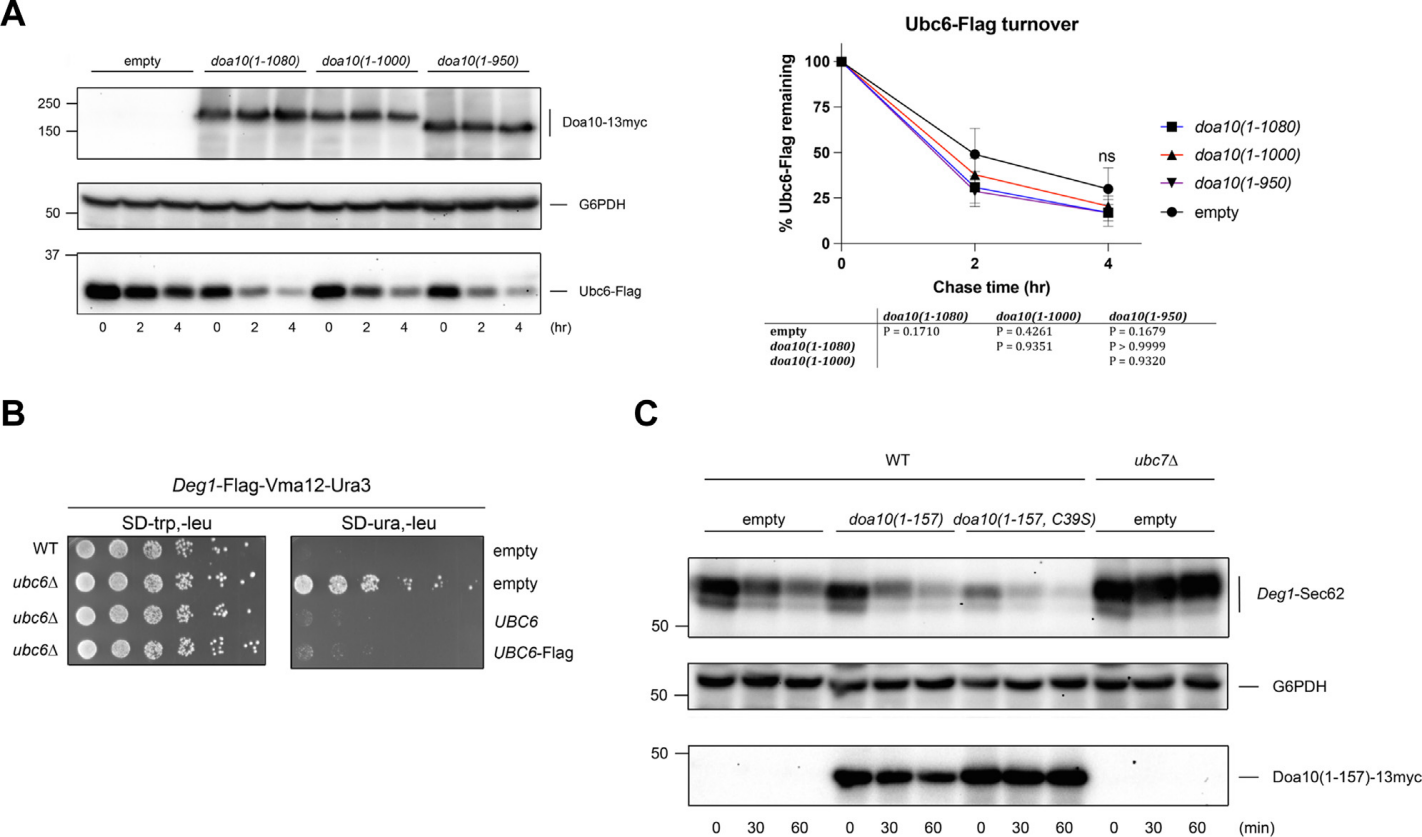
**Ectopic RING activity at the ER does not broadly inhibit ERAD.***A*, CHX-chase analysis of the Doa10 substrate Ubc6-Flag was performed as in Figure 1*D*, except cells carried pRS416-*UBC6*-Flag and the indicated p414GPD-based *doa10* alleles. Molecular size markers (in kDa) are shown at *left*. *B*, growth assays with *Deg1*-Flag-Vma12-Ura3 expressed from p414MET25. MHY500 cells were cotransformed with pRS315 or the indicated pRS315-UBC6 plasmid. *C*, CHX-chase analysis of the Hrd1 substrate *Deg1*-Sec62 in MHY500 or MHY551 (*ubc7Δ)* cells transformed with p416MET25-*Deg1*-Flag-Sec62-2xProtA and the indicated overexpressed Doa10 fragments. Molecular size markers (in kDa) are shown at *left*. This experiment was performed twice. ERAD, endoplasmic reticulum–associated degradation; CHX, cycloheximide.

To determine whether increased RING activity at the ER inhibits all ERAD, we investigated the turnover of substrates from different ERAD classes. This included the E2 Ubc6, a Doa10 ERAD-M substrate that is likely recognized and ubiquitylated through unique mechanisms (8, 14, 31). Unlike degradation of Doa10 ERAD-C substrates, Ubc6-Flag turnover proceeded unabated in cells overexpressing Doa10 truncation mutants (Fig. 3*A*). Ubc6-Flag complemented *ubc6Δ* cells for membrane ERAD-C turnover, indicating proper TM insertion and interaction with the Doa10 complex (Fig. 3*B*). Turnover of *Deg1*-Sec62, an ERAD-T substrate (translocon-associated) of Hrd1 that aberrantly engages the Sec61 translocon (32, 33), was similarly unaffected by *doa10(1-157)* overexpression (Fig. 3*C*). These results indicate ectopic RING activity at the ER does not broadly inhibit ERAD.

### Ubc6 can be degraded independently of the known ERAD E3 complexes and Dfm1

**Figure 4:**
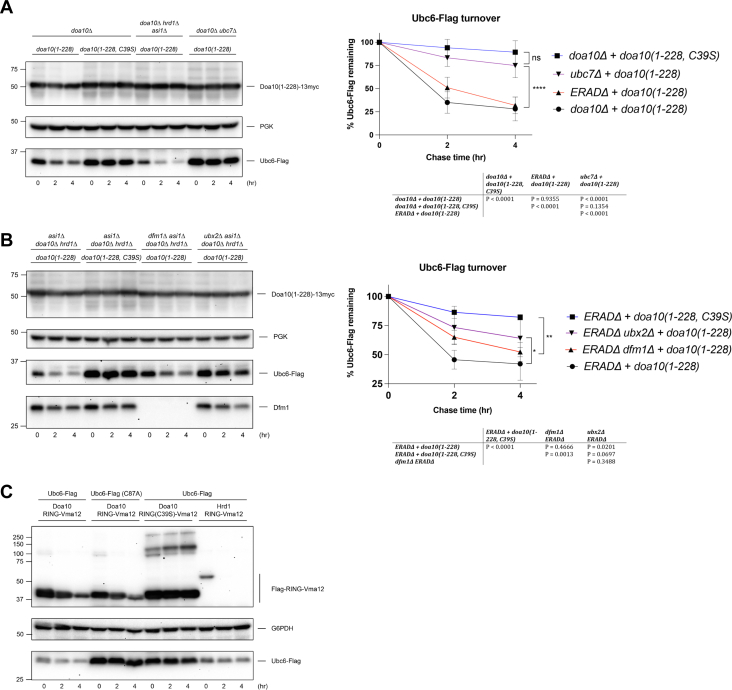
**Ubc6 can be degraded independently of the known ERAD E3 complexes and Dfm1.***A*, CHX-chase analysis of Ubc6-Flag turnover in the indicated strains with Doa10 mutant overexpression. Experiments were performed in MHY1685 (*doa10Δ*), MHY11132 (*doa10Δ hrd1Δ asi1Δ*), or MHY12228 (*doa10Δ ubc7Δ*) cells transformed with pRS416-*UBC6*-Flag and the indicated p414GPD-based *doa10* alleles. Ubc6-Flag band intensities were normalized to PGK levels. Molecular size markers (in kDa) are shown at *left*. *B*, CHX-chase analysis of Ubc6-Flag turnover upon Doa10 mutant overexpression in MHY11132 (*doa10Δ hrd1Δ asi1Δ*), MHY12461 (*doa10Δ hrd1Δ asi1Δ dfm1Δ*), and MHY12451 (*doa10Δ hrd1Δ asi1Δ ubx2Δ*) cells. Molecular size markers (in kDa) are shown at *left*. *C*, CHX-chase analysis of Ubc6-Flag turnover during RING-Vma12 overexpression was performed as in *B* in MHY11132 (*doa10Δ hrd1Δ asi1Δ*). Molecular size markers (in kDa) are shown at *left*. This experiment was performed three times. ERAD, endoplasmic reticulum–associated degradation; CHX, cycloheximide. ∗*p* < 0.05; ∗∗*p* < 0.01; ∗∗∗∗*p* < 0.0001.

While the turnover kinetics of Ubc6-Flag were not slowed by Doa10 mutant overexpression, its steady-state levels were significantly lower in these cells (Figs. 3*A* and S2), suggesting Ubc6 turnover might actually be enhanced under these conditions. We therefore tested whether the dominant-negative mutants could cause degradation of Ubc6-Flag and whether this required endogenous Doa10. We found the *doa10(1-228)* mutant targeted Ubc6-Flag for degradation in cells lacking *DOA10* or even lacking all three ERAD E3 complexes (*ERADΔ*) (Figs. 4*A* and S3). Efficient degradation of Ubc6-Flag by *doa10(1-228)* required RING activity, Ubc7, and the Cdc48 cofactor Ubx2, whereas the rhomboid pseudoprotease Dfm1 was dispensable (Fig. 4, *A* and *B*). These results were unexpected since similar or longer Doa10 mutants are incapable of degrading Ubc6 when expressed at endogenous levels (14). The *dfm1Δ ERADΔ* quadruple mutant lacks all recently proposed retrotranslocation channels; nevertheless, when overexpressed, *doa10(1-228)* still stimulated Ubc6-Flag degradation.

Next, we investigated whether other dominant-negative constructs could similarly reduce Ubc6-Flag levels by mediating its turnover. Indeed, we observed less Ubc6-Flag when overexpressing the Doa10 or Hrd1 RING-Vma12 proteins in the *ERADΔ* mutant; however, increased turnover of Ubc6-Flag was less evident due to the instability of the RING-Vma12 proteins (Fig. 4*C*, top panel). The Doa10 RING-Vma12 protein is stable when it contains an inactivated RING domain (C39S); therefore, the RING-Vma12 chimeras are likely “self-ubiquitylating substrates” (SUSs) (34). Together, these data suggest the dominant-negative effect towards ERAD-C substrates of Doa10 could be due to reduced levels of active Ubc6, an E2 required for the ubiquitylation of these substrates (14).

### Increased Ubc6 levels rescue ERAD-C from Doa10 mutant overexpression

**Figure 5:**
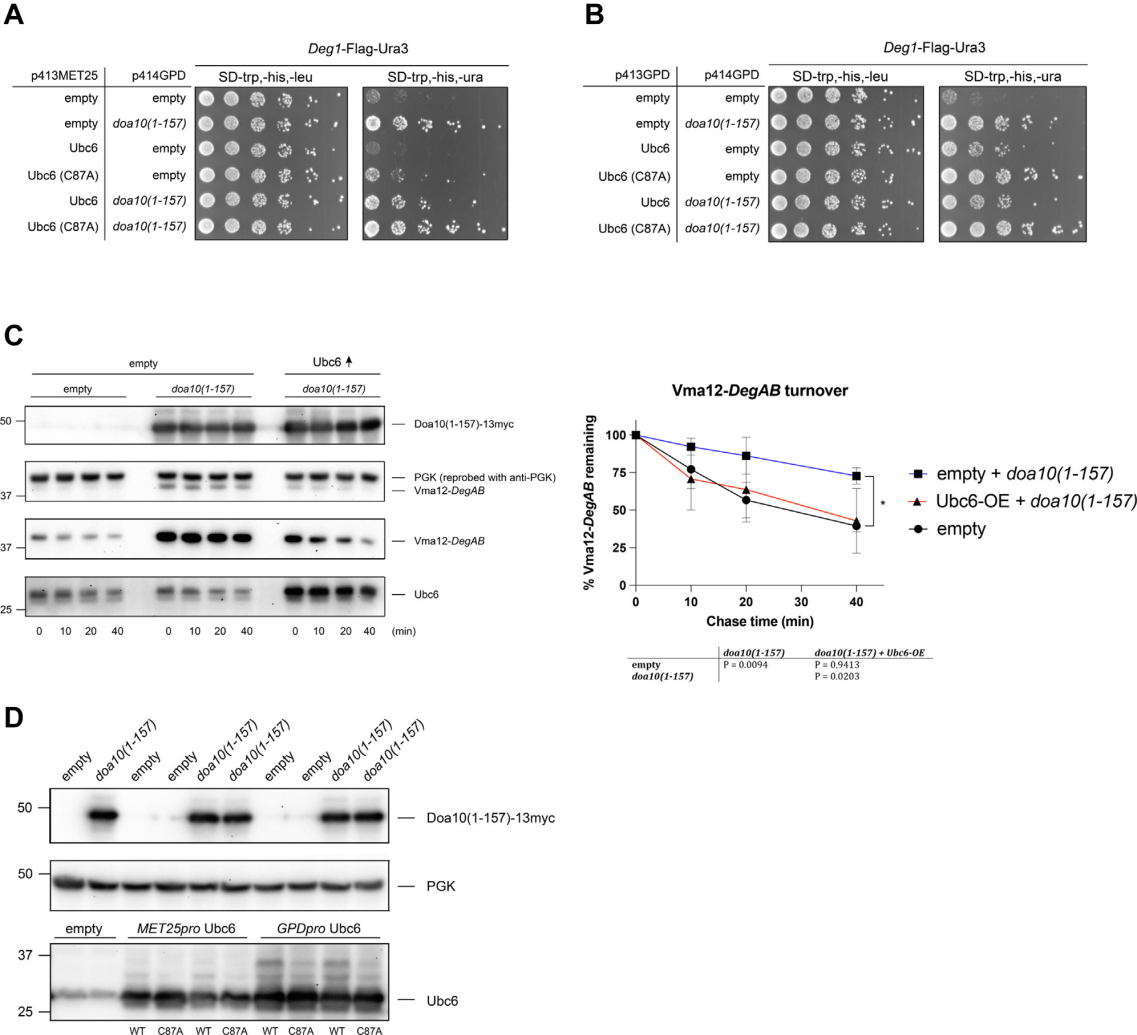
**Increased Ubc6 levels rescue ERAD-C from Doa10 mutant overexpression.***A*, growth assays with the soluble *Deg1*-Flag-Ura3 reporter expressed from p415MET25. MHY500 cells were also transformed with p414GPD-doa10(1-157) or empty vector and the p413MET25-Ubc6 derivatives. This experiment was performed twice. *B*, *Deg1*-Flag-Ura3 was analyzed as in panel A except with the indicated p413GPD-Ubc6 derivatives. This experiment was performed twice. *C*, CHX-chase analysis of Vma12-*DegAB* turnover during doa10(1-157) and Ubc6 cooverexpression was performed as in Figure 1*E*, except MHY500 cells were transformed with p414GPD or p414GPD-doa10(1-157) and p413MET25 (empty or with *UBC6*). Molecular size markers (in kDa) are shown at *left*. *D*, expression analysis of doa10(1-157) and Ubc6 from (*A* and *B*). Molecular size markers (in kDa) are shown at *left*. ERAD, endoplasmic reticulum–associated degradation; CHX, cycloheximide.

The apparent correlation between reduced Ubc6-Flag and ERAD-C inhibition led us to speculate that Ubc6 becomes limiting during Doa10 mutant or RING-Vma12 overexpression (Fig. 4). We used the degradation-sensitive growth assay to monitor *Deg1*-Flag-Ura3 turnover in cells simultaneously overexpressing *doa10(1-157)* and *UBC6*. Overexpression of *UBC6* from either the *MET25* or *GPD* promoter modestly rescued *Deg1*-Flag-Ura3 turnover during *doa10(1-157)* overexpression (Fig. 5, *A* and *B*), supporting the notion that Ubc6 levels are limiting under these conditions. Consistent with our previous findings (35), strong overproduction of the Ubc6 protein had the opposite effect—*Deg1*-Flag-Ura3 stabilization. These results could explain why we were unable to observe full rescue of *Deg1*-based turnover when overexpressing *UBC6*.

We directly tested if increasing Ubc6 levels rescued turnover of another ERAD-C substrate in cells with high Doa10 mutant levels. CHX-chase analysis of Vma12-*DegAB* degradation in cells overexpressing both *UBC6* and *doa10(1-157)* revealed restoration of normal degradation kinetics (Fig. 5*C*). Together, these data indicate that Ubc6 is limiting in cells with increased RING activity at the ER membrane even though overall Ubc6 levels are relatively unchanged in cells overexpressing *doa10(1-157)* (Fig. 5*D*).

## Discussion

Overexpression of ERAD factors is commonly used to study different aspects of ERAD in yeast and mammalian cells, including substrate identification and substrate retrotranslocation (19, 20, 21, 22). ERAD components are also upregulated to mitigate the accumulation of misfolded proteins during ER stress, an inherit feature of multiple human diseases (23, 36). Increased levels of the human orthologs of Doa10 and Hrd1 have been associated with poor cancer outcomes (24, 25, 26). Despite this, how ERAD E3 overexpression impacts general protein degradation at the ER is unclear. Importantly, we find that overexpression of full-length Hrd1 stabilizes non-Hrd1 substrates, suggesting unanticipated “off-target” changes in protein homeostasis under such artificial conditions and potentially during the aforementioned physiological perturbations.

In this study, we investigated the effects of E3 mutant overexpression on ERAD in budding yeast. Overexpression of several Doa10 mutants, including the minimal *doa10(1-157)* allele, inhibits ERAD-C substrate turnover (Fig. 1). This inhibitory effect arises from either increased Doa10 or Hrd1 RING activity at the ER membrane (Fig. 2). Under these conditions, Ubc6, a cognate E2 for Doa10 in ERAD-C (14), becomes limiting.

Exactly how Ubc6 becomes limiting during Doa10 mutant overexpression is uncertain. Because steady-state levels of Ubc6 remain relatively unchanged in cells overexpressing *doa10(1-157)*, it is unlikely ERAD-C inhibition is solely due to reduced bulk levels of Ubc6 (Fig. 5, *C* and *D*). The dominant-negative fusions of Vma12 to either the Doa10 or Hrd1 RING domain lack the minimal E2-binding region of Doa10; nevertheless, these RING domains can interact very weakly with Ubc6 (14, 37), and their strong overexpression will promote E2 interaction, potentially enabling the Doa10 mutants and RING chimeras to titrate Ubc6 from endogenous Doa10. This titration model would explain why increased Doa10 or Hrd1 RING activity at the ER membrane specifically inhibits Doa10-mediated ERAD-C, which requires Ubc6 (14).

Previous studies have demonstrated the utility of chimeric SUSs for investigating ERAD E3-independent retrotranslocation (34, 38). Here, we characterize additional SUSs that can be degraded independently of all ERAD E3 complexes in yeast (Fig. 4*C*). It will be interesting to test potential differences between these chimeras, which contain distinct ER TMs. The Vma12-containing SUSs defined here contain two TMs, as opposed to the eight TMs in the Hmg1-based SUS (34). Moreover, we characterized overexpression mutants of Doa10 that lack the regions responsible for protein channel formation and retrotranslocase activity *in vitro*; these degrade Ubc6-Flag independently of the ERAD E3 complexes and Dfm1 (14, 39). These results were unexpected because the degradation of Ubc6 requires Cdc48, an ATPase required for retrotranslocation, and Ubc6 has been used as a model substrate to study ERAD E3-mediated retrotranslocation *in vitro* (15, 39). Ubc6 localizes throughout the ER membrane, including the INM, so a novel retrotranslocation pathway may contribute to its turnover, potentially at the INM (6, 40). It is also possible Cdc48 and/or proteasomes directly mediate the retrotranslocation of certain ERAD substrates (41), such as those with 1 to 2 TMs. The overexpression constructs described here will be useful for testing these possibilities.

In summary, we have provided insights into the molecular consequences of ERAD E3 overexpression in budding yeast. We anticipate mammalian ERAD E3 overexpression has a similar impact, whereby ER-resident E2s become limiting. An important question is whether ERAD E2s become limiting in certain ERAD-related diseases or conditions of ER stress where ERAD E3s are upregulated.

## Experimental procedures

### Yeast methods

Yeast strains and plasmids used in this study are listed in Tables S1 and S2. Yeast were genetically manipulated using standard techniques and grown at 30 °C in minimal (SD) media (42). For the spot growth assays, cells were grown overnight in SD media (ura+), diluted to an A_600_ of 0.2, and serially diluted in 5-fold steps in water. Cells were spotted onto different plates and incubated for 2 to 3 days.

As *DOA10* is toxic to *Escherichia coli*, plasmids expressing *doa10(1-609)* or larger were cloned into p414*GPD* using an improved yeast gap repair protocol (43). The pRS416-Ubc6-Flag plasmid, which contains an internal Flag tag at the same location as HA-tagged *UBC6* (44)*,* was made by gap repair cloning. Other plasmids in this study were generated by restriction digestion and ligation or Quikchange mutagenesis. All overexpression mutants were verified by sequencing and immunoblotting.

### Cell extract preparation and immunoblotting

Cell extracts for expression analysis were prepared as described (14). Briefly, yeast were grown in selective SD media to mid-exponential phase, whereafter 2.5 A_600_ units were harvested and lysed by an alkaline lysis method (45). Lysates were resuspended in SDS sample buffer and incubated at 37 °C for 30 min, centrifuged to remove cell debris, and analyzed by SDS-PAGE and immunoblotting.

Immunoblotting was performed as described (46) with the following antibodies: rabbit anti-Doa10 at 1:2000 (27); mouse anti-MYC (9E10, Covance) at 1:10,000; rabbit anti-G6PDH (A9521, Sigma) at 1:10,000; mouse anti-FLAG (F3165; Sigma) at 1:2000 or 1:10,000; peroxidase anti-peroxidase (Sigma) at 1:1000; mouse anti-PGK (459250, Thermo Fisher Scientific) at 1:20,000; rabbit anti-Dfm1 at 1:2000 (47); rabbit anti-Ubc6 at 1:2000 (31). Primary antibody incubations were followed by peroxidase-coupled sheep anti-mouse or peroxidase-coupled goat anti-rabbit secondary antibodies (GE Healthcare) at 1:10,000 and visualized by enhanced chemiluminescence (48) on a G:Box system (Syngene).

### CHX-chase assays and protein extraction

CHX-chase assays were performed as described (14, 45). For quantification, band intensities from immunoblotting were quantified using Gene Tools (Syngene). Statistical analysis was performed using GraphPad Prism, where two-way ANOVA with Tukey’s post hoc analysis was applied for data comparison. All graphs represent data from three experiments and error bars represent ± SD. Significance is indicated as following: ns, not significant; ∗*p* < 0.05; ∗∗*p* < 0.01; ∗∗∗*p* < 0.001; ∗∗∗∗*p* < 0.0001.

## Data availability

All data are contained within the article.

## Supporting information

This article contains supporting information (49, 50, 51, 52).

## Conflict of interest

The authors declare that they have no conflicts of interest with the contents of this article.
