# Supplementary material for: Ectopic RING activity at the ER membrane differentially impacts ERAD protein quality control pathways
Source: J Biol Chem. 2023 Jan 19;299(3):102927. doi: 10.1016/j.jbc.2023.102927 (PMC9950527; doi:10.1016/j.jbc.2023.102927)
Supplement: Supplemental data [file mmc1.docx]

**
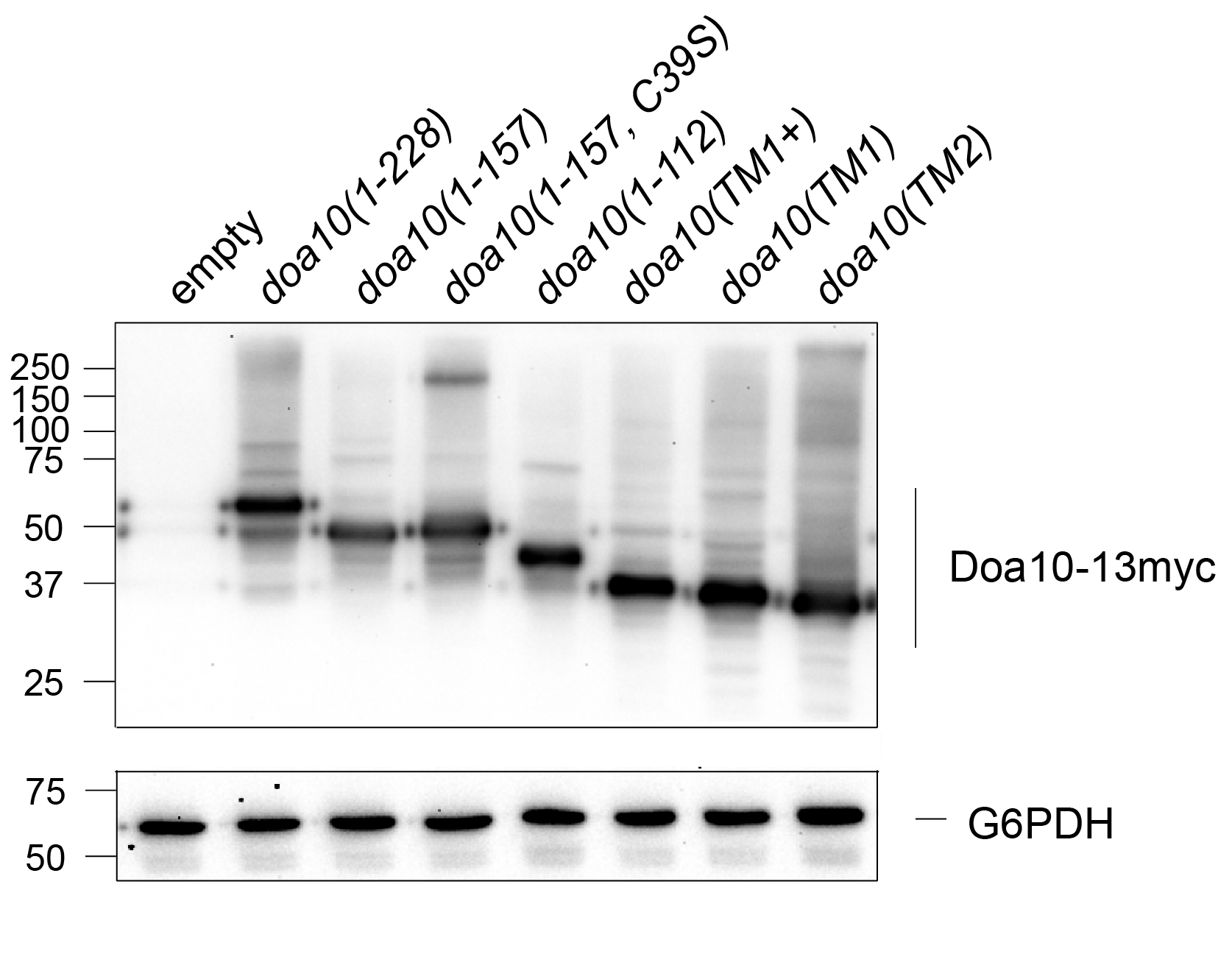
**

**Figure S1. Steady-state levels of the indicated Doa10 mutant proteins.**

Expression analysis of the indicated p414GPD-based *doa10* alleles from Figs. 2A and 2B. Lysates were analyzed by anti-MYC and anti-G6PDH immunoblotting. Molecular size markers (in kDa) are shown at left.


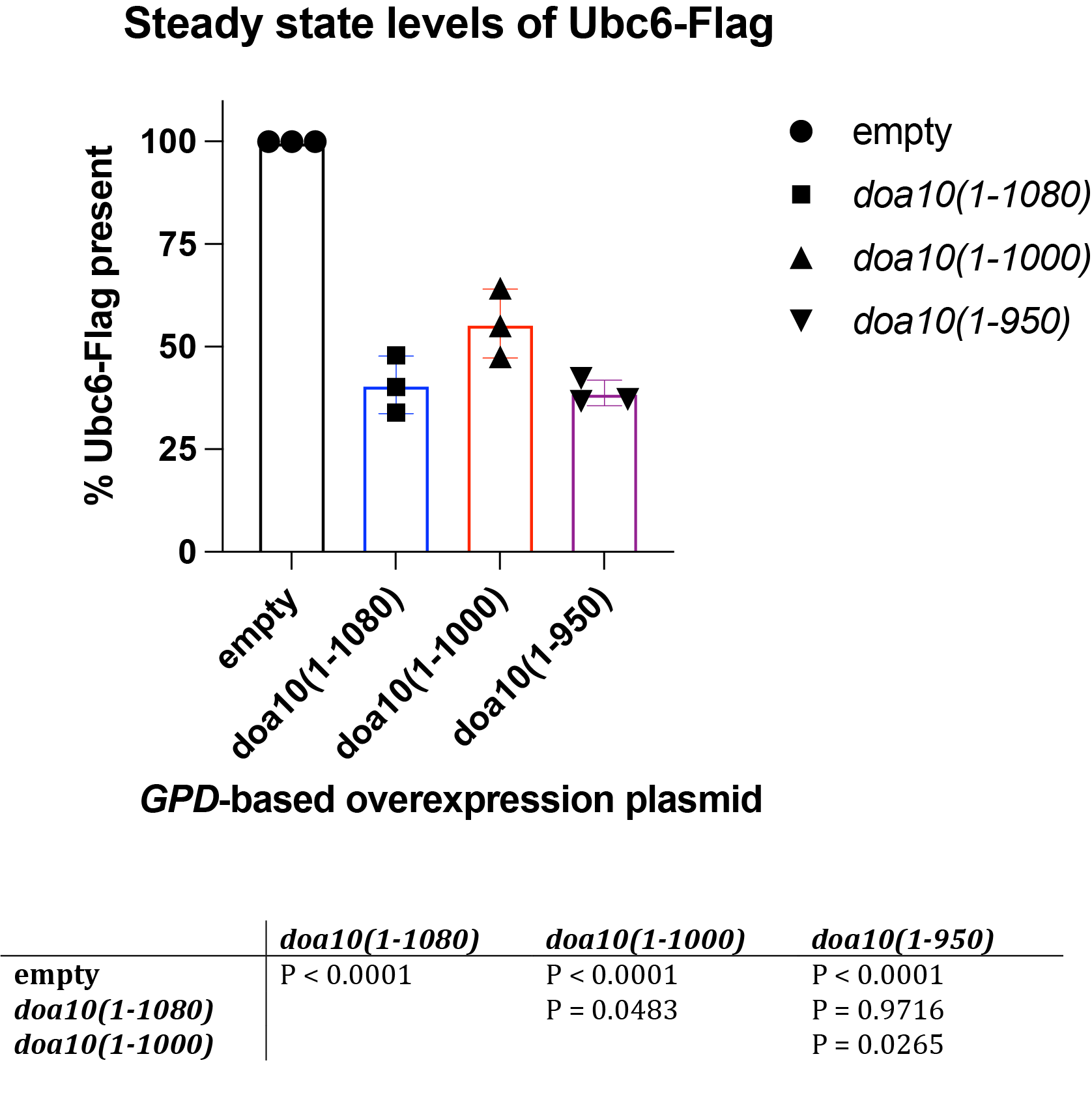


**Figure S2. Comparison of Ubc6-Flag steady-state levels upon Doa10 mutant overexpression.**

Analysis of Ubc6-Flag steady-state levels from Fig. 3A. Ubc6-Flag band intensities at time-point 0 were normalized to the G6PDH loading control. Data are represented as percentage of Ubc6-Flag levels in cells with the indicated p414-GPD-based *doa10* alleles mutants compared to the empty vector control. Mean values and SD (error bars) are shown. Statistical analysis was performed using ordinary one-way ANOVA with Tukey’s post hoc analysis.

**
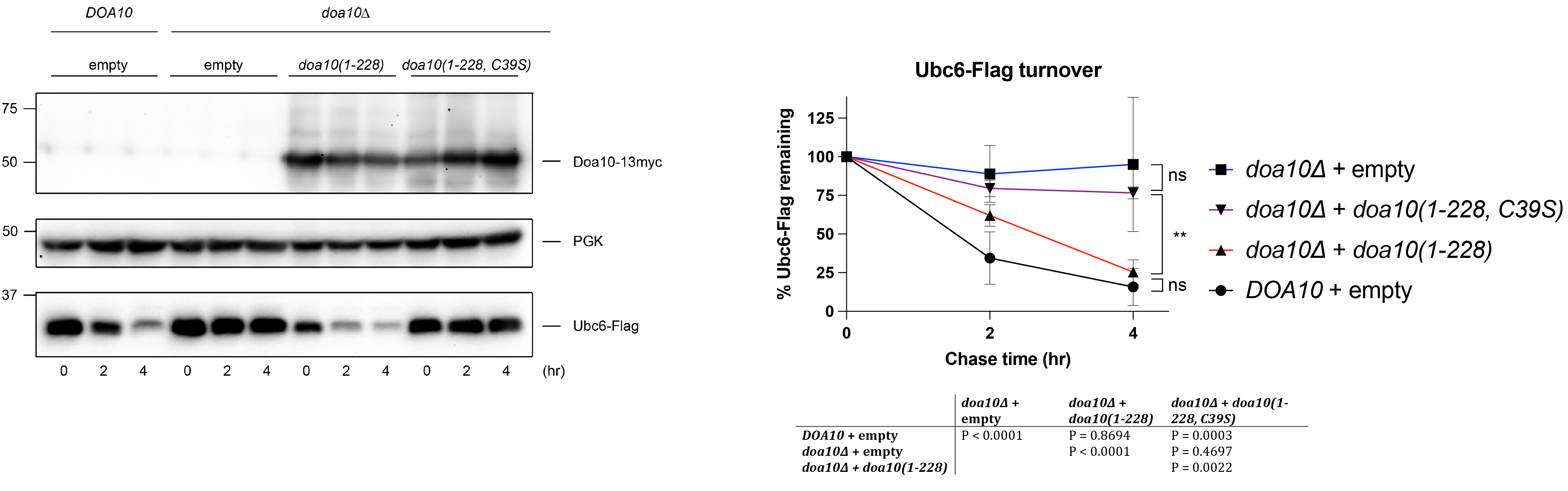
**

**Figure S3. Degradation rate of Ubc6-Flag is stimulated comparably by endogenous Doa10 and overexpressed Doa10(1-228).**

CHX-chase analysis of Ubc6-Flag turnover in the indicated strains with Doa10 mutant overexpression. Experiments were performed in MHY500 (*DOA10*) or MHY1685 (*doa10∆*) cells transformed with pRS416-Ubc6-Flag and the indicated p414GPD-based *doa10* alleles. Ubc6-Flag band intensities were normalized to PGK levels. Molecular size markers (in kDa) are shown at left. The graph (right panel) represents data as mean ± SD from three experiments.
