# Supplementary material for: Ectopic RING activity at the ER membrane differentially impacts ERAD protein quality control pathways
Source: J Biol Chem. 2023 Jan 19;299(3):102927. doi: 10.1016/j.jbc.2023.102927 (PMC9950527; doi:10.1016/j.jbc.2023.102927)
Supplement: Supplemental tables [file mmc2.docx]

**TABLES**

***Table S1. Yeast strains used in this study.***

| **Name** | **Alias** | **Genotype** | **Source** | **Figure** |
| --- | --- | --- | --- | --- |
| MHY500 |  | *MATa his3-∆200 leu2-3,112 ura3-52 lys2-801 trp1-1 gal2* | (49) | 1B, 1C, 1E, 2B, 2D, 2E, 2F, 3A, 3B, 3C, 5A, 5B, 5C, 5D, S1, S2 |
| MHY8655 |  | *MATa his3-∆200 leu2-3,112 ura3-52 lys2-801 trp1-1 gal2 DOA10-13myc::HIS3MX6* | (28) | 1B |
| MHY1685 |  | *MATa his3-∆200 leu2-3,112 ura3-52 lys2-801 trp1-1 gal2 doa10Δ::HIS3* | (2) | 1B, 4A |
| MHY11400 | ABM559 | *MATa his3-∆200 leu2-3,112 ura3-52 lys2-801 trp1-1 gal2 p414-GPD-DOA10-13myc* | This study | 1B, 1C, 1D |
| MHY11401 | ABM560 | *MATa his3-∆200 leu2-3,112 ura3-52 lys2-801 trp1-1 gal2 p414-GPD-doa10(1-1080)* | This study | 1C, 1D, 3A, S2 |
| MHY11402 | ABM561 | *MATa his3-∆200 leu2-3,112 ura3-52 lys2-801 trp1-1 gal2 p414-GPD-doa10(1-1000)* | This study | 1C, 1D, 3A, S2 |
| MHY11403 | ABM562 | *MATa his3-∆200 leu2-3,112 ura3-52 lys2-801 trp1-1 gal2 p414-GPD-doa10(1-950)* | This study | 1C, 1D, 3A, S2 |
| MHY11406 | ABM565 | *MATa his3-∆200 leu2-3,112 ura3-52 lys2-801 trp1-1 gal2 p414-GPD-doa10(1-609)* | This study | 1C |
| MHY496 |  | *MATa his3-∆200 leu2-3,112 ura3-52 lys2-801 trp1-1 gal2 ubc6Δ1::HIS3* | (49) | 3B |
| MHY551 |  | *MATa his3-∆200 leu2-3,112 ura3-52 lys2-801 trp1-1 gal2 ubc7Δ::LEU2* | (2) | 3C |
| MHY11132 | ABM297 | *MATa his3-∆200 leu2-3,112 ura3-52 lys2-801 trp1-1 gal2 doa10*Δ*::HIS3 hrd1∆::LEU2 asi1∆::kanMX6* | This study | 4A, 4B, 4C |
| MHY12228 | ABM654 | *MATa his3-∆200 leu2-3,112 ura3-52 lys2-801 trp1-1 gal2 doa10*Δ*::HIS3 ubc7Δ::LEU2* | This study | 4A |
| MHY12461 | ABM708 | *MATa his3-∆200 leu2-3,112 ura3-52 lys2-801 trp1-1 gal2 doa10*Δ*::HIS3 hrd1∆::LEU2 asi1∆::kanMX6 dfm1∆::natMX4* | This study | 4B |
| MHY12451 | ABM698 | *MATa his3-∆200 leu2-3,112 ura3-52 lys2-801 trp1-1 gal2 doa10*Δ*::HIS3 hrd1∆::LEU2 asi1∆::kanMX6 ubx2*Δ*::HIS3* | This study | 4B |
| MHY2923 |  | *MATa his3-∆200 leu2-∆1 ura3-52 lys2-801 trp1-∆63 DOA10-13myc::HIS3MX6* | (9) | Strain making |
| MHY10832 | ABM191 | *MATa his3-∆200 leu2-∆1 ura3-52 lys2-801 trp1-∆63 doa10 1-1080-13myc::HIS3MX6* | (14) | Strain making |
| MHY10842 | ABM201 | *MATa his3-∆200 leu2-∆1 ura3-52 lys2-801 trp1-∆63 doa10 1-1000-13myc::HIS3MX6* | (14) | Strain making |
| MHY2935 |  | *MATa his3-∆200 leu2-∆1 ura3-52 lys2-801 trp1-∆63 doa10 1-950-13myc::HIS3MX6* | (14) | Strain making |
| MHY2942 |  | *MATa his3-∆200 leu2-∆1 ura3-52 lys2-801 trp1-∆63 doa10 1-609-13myc::HIS3MX6* | S. Kreft (Hochstrasser Lab) | Strain making |
| MHY9130 |  | *MATa his3-∆200 leu2-∆1 ura3-52 lys2-801 trp1-∆63 ade2-101 doa10 1-228-13myc::HIS3MX6* | J. Berk (Hochstrasser Lab) | Plasmid making |
| MHY9202 |  | *MATa his3-∆200 leu2-∆1 ura3-52 lys2-801 trp1-∆63 ade2-101 doa10 1-157-13myc::HIS3MX6* | J. Berk (Hochstrasser Lab) | Plasmid making |

***Table S2. Plasmids used in this study.***

| **Name** | **Alias** | **Yeast selection marker** | **Yeast plasmid type** | **Description** | **Source** | **Figure** |
| --- | --- | --- | --- | --- | --- | --- |
| p414-*GPD* |  | *TRP1* | CEN | Empty vector control | (50) | 1B, 1C, 1E, 2B, 2D, 2E, 2F, 3A, 3C, 5A, 5B, 5C, 5D, S1, S2 |
| p414*-GPD-doa10(1-228)* |  | *TRP1* | CEN | *doa10(1-228)-13myc* driven by *GPD* promoter | This study | 1C, 2B, 2D, 4A, 4B, S1 |
| p415-*MET25*-*Deg1*-FL-Ura3 |  | *LEU2* | CEN | *Deg1*-Flag-Ura3 driven by *MET25* promoter | (51) | 1C, 2B, 2D, 2E, 5A, 5B, S1 |
| p414*-GPD-doa10(1-157)* |  | *TRP1* | CEN | *doa10(1-157)-13myc* driven by *GPD* promoter | This study | 1C, 1E, 2B, 2D, 3C, 5A, 5B, 5C, 5D, S1 |
| p415-*GPD*-*Deg1*-FL-Ura3 |  | *LEU2* | CEN | *Deg1*-Flag-Ura3 driven by *GPD* promoter | This study | 1D |
| p414*-GPD-doa10(1-157, C39S)* |  | *TRP1* | CEN | *doa10(1-157, C39S)-13myc* driven by *GPD* promoter | This study | 1E, 2B, 2D, 3C, S1 |
| p416*-GPD-FL-Vma12-pHIS-DegAB* |  | *URA3* | CEN | Flag-Vma12-pHIS-*DegAB* driven by *GPD* promoter | This study | 1E, 5C |
| p414*-GPD-doa10(1-112)* |  | *TRP1* | CEN | *doa10(1-112)-13myc* driven by *GPD* promoter | This study | 2B, S1 |
| p414*-GPD-doa10(101-157)* | *doa10(TM1+)* | *TRP1* | CEN | *doa10(101-157)-13myc* driven by *GPD* promoter | This study | 2B, S1 |
| p414*-GPD-doa10(122-157)* | *doa10(TM1)* | *TRP1* | CEN | *doa10(122-157)-13myc* driven by *GPD* promoter | This study | 2B, S1 |
| p414*-GPD-doa10(190-228)* | *doa10(TM2)* | *TRP1* | CEN | *doa10(190-157)-13myc* driven by *GPD* promoter | This study | 2B, S1 |
| p414*-GPD-FL-doa10-RING-Vma12* |  | *TRP1* | CEN | Flag-Doa10 RING (1-112)-Vma12 driven by *GPD* promoter | This study | 2D, 2E, 2F, 4C |
| p414*-GPD-FL-doa10-RING-C39S-Vma12* |  | *TRP1* | CEN | Flag-Doa10 RING (C39S)-Vma12 driven by *GPD* promoter | This study | 2D, 2E, 2F, 4C |
| p414*-FL-Vma12-pHIS* |  | *TRP1* | CEN | Flag-Vma12-pHIS driven by *GPD* promoter | This study | 2D |
| p414*-GPD-FL-hrd1-RING-Vma12* |  | *TRP1* | CEN | Flag-Hrd1 RING (320-531)-Vma12 driven by *GPD* promoter | This study | 2E, 2F, 4C |
| p414*-GPD-FL-hrd1-RING-C399S-Vma12* |  | *TRP1* | CEN | Flag-Hrd1 RING (C399S)-Vma12 driven by *GPD* promoter | This study | 2E, 2F |
| p414*-GPD-FL-asi1-RING-Vma12* |  | *TRP1* | CEN | Flag-Asi1 RING (473-624)-Vma12 driven by *GPD* promoter | This study | 2E, 2F |
| p414*-GPD-FL-asi1-RING-C583S-Vma12* |  | *TRP1* | CEN | Flag-Asi1 RING (C583S)-Vma12 driven by *GPD* promoter | This study | 2E, 2F |
| p414*-GPD-FL-asi1-RING-C585S-Vma12* |  | *TRP1* | CEN | Flag-Asi1 RING (C585S)-Vma12 driven by *GPD* promoter | This study | 2E, 2F |
| p424-*GPD*-*HRD1* |  | *TRP1* | 2µ | Hrd1 driven by *GPD* promoter | This study | 2E |
| pRS416-*UBC6*-Flag |  | *URA3* | CEN | Ubc6-Flag (internal tag) driven by endogenous promoter | This study | 3A, 4A, 4B, 4C, S2 |
| p414-*MET25*-*Deg1*-FL-Vma12-Ura3 |  | *TRP1* | CEN | Deg1-Flag-Vma12-Ura3 driven by *MET25* promoter | This study | 3B |
| pRS315 |  | *LEU2* | CEN | Empty vector control | This study | 3B |
| pRS315-*UBC6* |  | *LEU2* | CEN | Ubc6 driven by endogenous promoter | This study | 3B |
| pRS315 *UBC6*-Flag |  | *LEU2* | CEN | Ubc6-Flag (internal tag) driven by endogenous promoter | This study | 3B |
| p416-*MET25*-*Deg1*-Sec62-ProtA |  | *URA3* | CEN | Deg1-Sec62-ProtA driven by *GPD* promoter | (32) | 3C |
| p414*-GPD-doa10(1-228, C39S)* |  | *TRP1* | CEN | *doa10(1-228, C39S)-13myc* driven by *GPD* promoter | This study | 4A, 4B |
| pRS416-*UBC6*-Flag (C87A) |  | *URA3* | CEN | Ubc6-Flag (C87A) driven by endogenous promoter | This study | 4C |
| p413-*MET25* |  | *HIS3* | CEN | Empty vector control | (52) | 5A, 5B, 5C, 5D |
| p413-*MET25-UBC6* |  | *HIS3* | CEN | Ubc6 driven by *MET25* promoter | This study | 5A, 5C, 5D |
| p413-*MET25-UBC6 (C87A)* |  | *HIS3* | CEN | Ubc6 (C87A) driven by *MET25* promoter | This study | 5A, 5D |
| p413-*GPD-UBC6* |  | *HIS3* | CEN | Ubc6 driven by *GPD* promoter | This study | 5B, 5D |
| p413-*GPD-UBC6 (C87A)* |  | *HIS3* | CEN | Ubc6 (C87A) driven by *GPD* promoter | This study | 5B, 5D |
